# Flame self-interactions in MILD combustion of homogeneous and inhomogeneous mixtures

**DOI:** 10.1038/s41598-024-55782-3

**Published:** 2024-03-06

**Authors:** Khalil Abo-Amsha, Hazem S. A. M. Awad, Nilanjan Chakraborty

**Affiliations:** School of Engineering, Newcastle University, Newcastle upon tyne, NE1 7RU UK

**Keywords:** Mechanical engineering, Computational science, Fluid dynamics

## Abstract

Flame self-interaction (FSI) events in Moderate or Intense Low-Oxygen Dilution (MILD) combustion of homogeneous and inhomogeneous mixtures of methane and oxidiser have been analysed using three-dimensional Direct Numerical Simulations (DNS). The simulations have been conducted at the same global equivalence ratio ($\langle \phi \rangle = 0.8$) for different levels of $\mathrm {O_2}$ concentration (dilution) and initial turbulence intensities. It has been reported that both homogeneous and inhomogeneous mixture MILD combustion cases exhibit significant occurrences of FSI events, with the peak frequency of FSI events occurring towards the burned gas side in all cases. Moreover, the frequency of FSI events increases with increasing dilution level and turbulence intensity, but the presence of mixture inhomogeneity leads to a reduction in total FSI events. In all cases, the cylindrical FSI topologies (i.e. tunnel formation and tunnel closure) were found to have a higher likelihood of occurrence compared to spherical FSI topologies (i.e. unburned and burned gas pockets). The geometries of FSI topologies were also analysed using the mean and Gaussian curvatures. It has been shown that the inward propagating spherical FSI topologies (i.e. unburned gas pockets) are associated with negative mean curvature, while outward propagating spherical FSI topologies (i.e. burned gas pockets) are associated with positive mean curvature. Moreover, tunnel formation (tunnel closure) FSI topologies predominantly exhibit either elliptic geometries with positive (negative) mean curvature or hyperbolic saddle geometries with negative (positive) mean curvature. It has been shown for the first time in MILD combustion that the mean values of kinematic restoration and dissipation terms in the transport equation of the magnitude of the reaction progress variable conditional upon the reaction progress variable tend to cancel each other in the vicinity of the critical points associated with cylindrical topologies. Thus, the singular contributions in these terms, which are obtained from analytical descriptions in the vicinity of tunnel formation and tunnel closure topologies, do not affect the balance equation of the magnitude of the gradient of the reaction progress variable. Consequently, there is no need for a separate model treatment for singularities in modelling approaches based on the magnitude of the gradient of the reaction progress variable. The FSI events in the reaction dominated and propagating flame regions of MILD combustion have also been analysed for the first time. It has been found that more FSI events occur in the reaction dominated region, particularly towards the burned gas side. However, the majority of spherical FSI topologies are found in the propagating flame region. The findings from this study indicate that turbulence intensity, dilution level and mixture inhomogeneity effects need to be considered in any attempt to extend flame surface-based modelling approaches to MILD combustion.

## Introduction

Moderate or Intense Low oxygen Dilution (MILD) combustion has the potential to improve thermal efficiency and reduce emissions^1–3^. Thus, it presents an attractive option for addressing the need to comply with increasingly stringent environmental regulations without compromising on energy conversion requirements.

In MILD combustion, the mixture’s initial temperature ($T_0$) is higher than the autoignition temperature of the mixture ($T_{ign}$) and the maximum temperature rise due to combustion is less than the mixture’s autoignition temperature (i.e. $\Delta T< T_{ign}$)^3^. Cavaliere and de Joannon^3^ provided an extensive review of both experimental and computational research findings on MILD combustion and important physical insights into this combustion technique. Plessing et al.^4^ investigated MILD combustion using Planar Laser-Induced Fluorescence images of OH radicals (OH-PLIF) and Rayleigh thermometry. The OH-PLIF images indicated the presence of flame fronts inside the combustor, whereas Rayleigh thermometry suggested a distributed combustion^4^. Qualitatively similar observations were also reported by Özdemir and Peters^5^ and Dally et al.^6^ based on their experimental investigations.

Several recent Direct Numerical Simulation (DNS) studies^7–13^ have been conducted to gain improved insights into the physics of MILD combustion, and it was revealed that the interaction between the thin reaction zones is principally responsible for the appearance of distributed combustion. Minamoto et al.^7^ compared homogeneous mixture MILD combustion with conventional premixed combustion using DNS data and observed that the reaction zones were highly convoluted under MILD combustion conditions. Minamoto et al.^7^ also suggested that $\mathrm {O_2}$ concentration influences the extent of the convolution and interaction of the reaction zones in MILD combustion. These aspects were subsequently confirmed for inhomogeneous mixture MILD combustion by Doan et al.^12,13^ using DNS data. Thus, Flame self-interaction (FSI) is an intrinsic feature of MILD combustion and the analysis of the local FSI topologies could potentially provide important physical insights into mutual annihilation and pocket burnout events under MILD combustion conditions. These events result in a rapid local flame surface area destruction, which significantly affect the surface area of the flame front and, consequently, the overall burning rate^5,12^. Therefore, an improved understanding of FSI events can potentially be helpful to the modelling of MILD combustion, particularly for closures that depend on the description of the flame surface.

A varity of methods have been employed for the identification of topological features in flames. These include: automatic feature extraction using complex wavelet transform^14^, Minkowski functional based technique^9,15^ and an approach leveraging the critical point theory^16–18^.

Minamoto et al.^9^ investigated the morphology of the reaction zone in MILD combustion using an approach based-on Minkowski functionals^15^. It was observed that the most common reaction zone structure has a pancake-like shape, which is consistent with experimental findings by Dally et al.^6^ The pancake shaped reaction structures were found to be the result of events such as autoignition, flames propagating into mixtures of reactants, products, intermediates and radicals, as well as the interactions between these flames^9^. Recently, Abo-Amsha and Chakraborty^19^ employed an approach based on the critical point theory to analyse FSI topologies in turbulent homogeneous mixture MILD combustion of n-heptane. It was found that the cylindrical, tunnel like-topologies have the highest frequency of all FSI topologies, and the peak FSI frequency occurred at $c \approx 0.5$. Abo-Amsha and Chakraborty^19^ also qualitatively assessed the effects of dilution level and initial turbulence intensity of the distribution on FSI events.

However, a direct comparison between FSI events, in terms of their frequencies and topologies, in homogeneous and inhomogeneous mixture MILD combustion for identical global mean equivalence ratio and turbulent flow conditions is yet to be conducted. Furthermore, a more quantitative assessment of the effects of dilution level (i.e. $\mathrm {O_2}$ concentration) and turbulence intensity on the FSI frequency in both homogeneous and inhomogeneous mixture MILD combustion is yet to be made. These gaps in the existing literature are addressed in this study by analysing three-dimensional DNS datasets of both homogeneous and inhomogeneous MILD combustion cases with different dilution levels (i.e. $X_{O2} = 4.8\%$ and $3.5\%$) and under different turbulence intensities. This work also analyses, for the first time under MILD combustion conditions, the evolution of the magnitude of the reactive scalar gradient conditionally averaged upon the reaction progress variable in the vicinity of FSI events, and the distribution of FSI topologies in the reaction dominated and propagating flame regions of MILD combustion. Thus, the findings of this analysis can help guide model development efforts in the context of MILD combustion, particularly for modelling approaches based on the magnitude of the reactive scalar gradient.

The main objectives of the present study are: 1. To investigate the differences in the distribution and frequency of FSI events between homogeneous and inhomogeneous mixture MILD combustion at the same global equivalence ratio. 2. To provide insights into the effects of dilution level and turbulence intensity on the frequency and distribution of FSI events in the reaction progress variable (*c*) space for both homogeneous and inhomogeneous mixture MILD combustion. 3. To check the boundedness of the balance equation for the magnitude of the reacting scalar gradient conditionally averaged upon *c* in the vicinity of FSI events through analysing the statistical behaviour of the kinematic restoration and dissipation terms in the balance equation. 4. To analyse the distribution and frequency of FSI events in both the reaction dominated and propagating flame regions of MILD combustion.

To achieve these objectives, a DNS dataset for turbulent MILD combustion has been analysed. The studied cases are briefly introduced in the next section. This is followed by a results section that combines descriptions of the mathematical background unpinning the proposed analysis, outcomes of applying the analysis to the DNS dataset, and the discussion of main findings. Following that, the main findings are summarised, and conclusions are drawn. Finally, the numerical implementation pertaining to this analysis is briefly presented in the final section of this paper.

## The studied cases

The thermochemical conditions for the laminar flames used to generate the initial and inflow fields for the DNS dataset are given in Table 1. These include the composition of the oxidiser stream, the resulting laminar flame speed ($S_L$) and thermal flame thickness ($\delta _{th}$) at an equivalence ratio $\phi =0.8$, and the initial temperature of the reactants ($T_0$). Here, the thermal flame thickness is defined as $\delta _{th}=(T_P-T_0)/ \max | \nabla T |_L$, where *T*, $T_P$ and $T_0$ are the instantaneous, products’ and reactants’ temperatures, respectively. The subscript “L” refers to the values in the corresponding 1D unstretched premixed flame. The laminar flame speed ($S_L$) and the thermal flame thickness ($\delta _{th}$) in Table 1 were reported for the equivalence ratio $\phi =0.8$ that is equal to the global mean equivalence ratio of the current inhomogeneous mixture simulations (i.e. $\langle \phi \rangle = 0.8$). The two levels of dilution (i.e. $\mathrm {O_2}$ concentration) considered here originate from the oxidiser stream compositions in Table 1. The low dilution cases (referred to by LD hereafter) have the oxygen mole fraction of $X_{O2} = 4.8\%$ in the oxidiser stream, while the high dilution cases (HD, hereafter) correspond to $X_{O2} = 3.5\%$ in the oxidiser stream.

The initial turbulence conditions for the current DNS dataset are shown in Table 2. These include the initial turbulence intensity ($u'$) and integral length scale ($\ell$) as well as the ratios $u'/S_L$ and $\ell /\delta _{th}$. In all cases but one, the initial turbulence intensities and integral length scales yield consistent values for the ratios $u'/S_L$ and $\ell /\delta _{th}$ as can be seen from Table 2. Thus, in the current analysis, the cases simulated at the lower turbulence intensity will be referred to by $u'/S_L \approx 4.0$, while the ones simulated at the higher turbulence intensity will be indicated with $u'/S_L \approx 8.0$.

The DNS simulations have been conducted using a skeletal methane-air chemical mechanism comprising 16 species and 25 reactions^20^. A complete description of the numerical procedure is given in the Methods section.

**Table 1:** The thermochemical conditions and the oxidiser stream compositions of the 1D laminar flames used to initialise the DNS scalar fields.

Case	$X_{O2}$ (%)	$X_{CO2}$ (%)	$X_{H2O}$ (%)	$X_{N2}$ (%)	$S_L|_{\phi = 0.8}$ (m/s)	$\delta _{th}|_{\phi = 0.8}$ (mm)	$T_0$ (K)
Low Dilution (LD)	4.8	6.1	12.1	75.1	3.2	0.62	1500
High Dilution (HD)	3.5	6.6	13.2	75.3	2.3	0.80	1500

**Table 2:** Initial turbulence intensities ($u'$) and length scales ($\ell$) for the cases considered.

	Homogeneous MILD				Inhomogeneous MILD			
	$u' \, (m/s)$	$\ell \, (mm)$	$u'/S_L$	$\ell /\delta _{th}$	$u' \, (m/s)$	$\ell \, (mm)$	$u'/S_L$	$\ell /\delta _{th}$
LD ($X_{O2}=4.8\%$)	12.8	1.55	4.0	2.5	12.8	1.55	4.0	2.5
	25.6	1.55	8.0	2.5	25.6	1.55	8.0	2.5
HD ($X_{O2}=3.5\%$)	9.20	2.0	4.0	2.5	12.8	1.55	5.6	1.9
	18.4	2.0	8.0	2.5	25.6	1.55	11.1	1.9

## Results

### Overview of the instantaneous fields

The extent of completion of the chemical reaction can be quantified in terms of a reaction progress variable (*c*), which increases monotonically from zero in the unburned gas to one in the fully burned products. The reaction progress variable (*c*) can be defined in terms of fuel mass fraction $Y_F$ in the following manner^21^:

1 $$\begin{aligned} c = \frac{\xi Y_{F\infty }-Y_F}{\xi Y_{F\infty } -\max \left[ 0, \frac{\xi - \xi _{st}}{1 - \xi _{st}}\right] Y_{F\infty }} \end{aligned}$$

where $Y_{F\infty }$ is the fuel mass fraction in the fuel stream (equals to one for a pure fuel stream). The mixture fraction ($\xi$) and its stoichiometric value ($\xi _{st}$) are defined in terms of the elemental mass fractions and atomic masses as follows^22^:

2 $$\begin{aligned} \begin{aligned} \xi&= \frac{2Z_C/W_C + 0.5 Z_H/W_H + (Z_{O,2}-Z_O)/W_O}{2 Z_{C,1}/W_C + 0.5 Z_{H,1}/W_H + Z_{O,2}/W_O} \\ \xi _{st}&= \frac{Z_{O,2}/W_O}{2 Z_{C,1}/W_C + 0.5 Z_{H,1}/W_H + Z_{O,2}/W_O} \end{aligned} \end{aligned}$$

where $Z_j$ and $W_j$ are the elemental mass fractions and atomic masses for carbon, oxygen, and hydrogen. The subscripts 1 and 2 refer to fuel and oxidiser streams, respectively. The equivalence ratio is then given in terms of the mixture fraction as:

3 $$\begin{aligned} \phi = \frac{\xi (1-\xi _{st})}{\xi _{st}(1-\xi )} \end{aligned}$$

**Figure 1:**
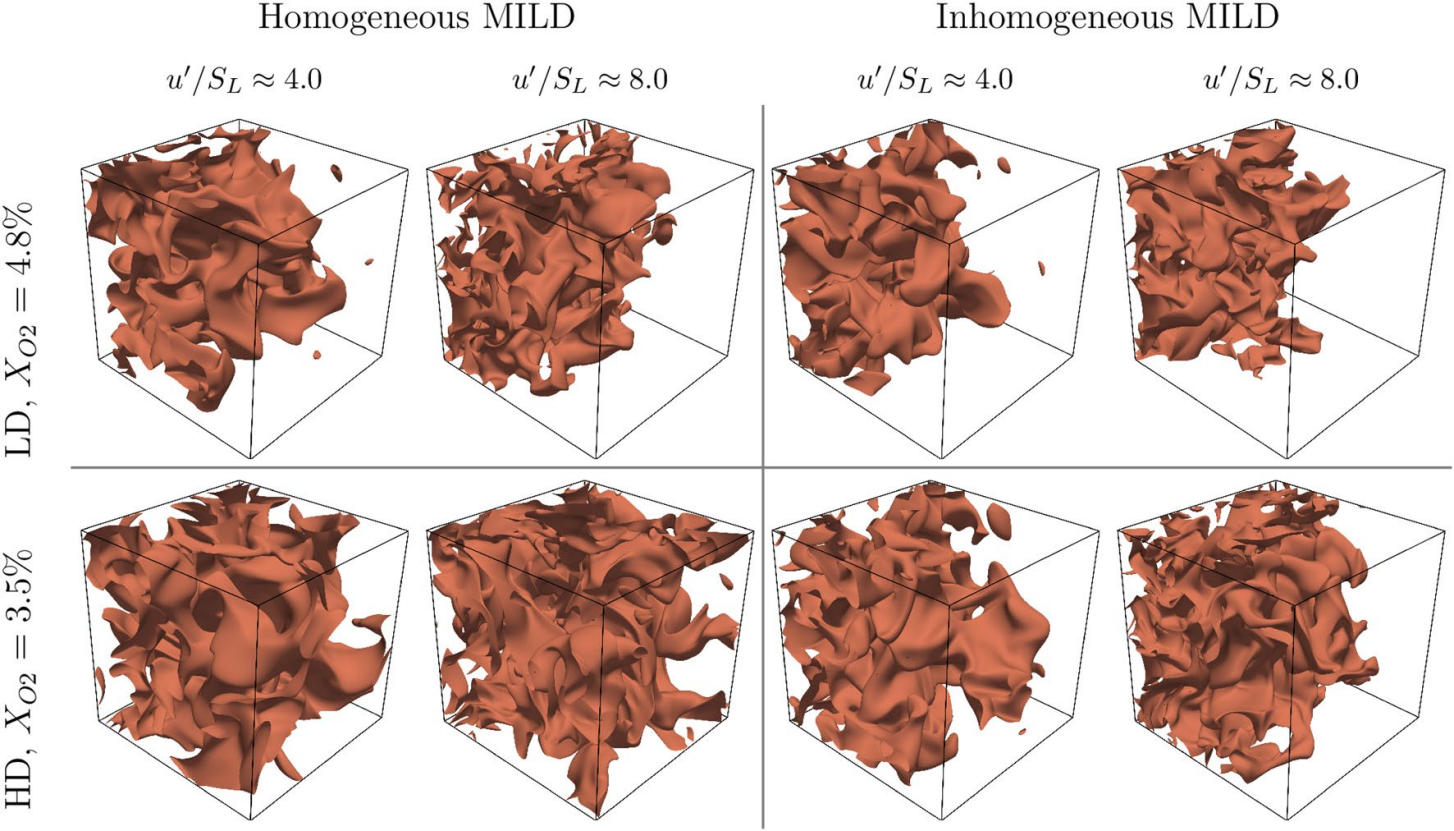
Isosurfaces of the reaction progress variable at $c=0.8$ in all cases considered. These *c* isosurfaces are considered as a marker of the flame surface for the current thermochemistry.

To provide a glimpse of the nature of MILD combustion, the instantaneous isosurfaces at $c=0.8$ are shown in Fig. 1. For the current thermochemistry, the maximum heat release occurs at $c \approx 0.8$ in the unstretched laminar premixed flames used to initialise the current simulations (see Table 1). Thus, the $c=0.8$ isosurface can be considered as a marker of the flame surface. Figure 1 gives the visual impression that the combustion process extends to about 50% of the computational domain in the low dilution (LD) cases, while it continues over most of the domain in the high dilution (HD) cases. This suggests a more distributed combustion in the high dilution cases. Moreover, Fig. 1 shows the strongly convoluted shape of the flame surface, which suggests a significant occurrence of FSI events throughout the reacting segment of the domain.

However, one cannot ascertain from Fig. 1 the effect of mixture inhomogeneity, dilution level, or turbulence intensity on the frequency of FSI events. Thus, a more comprehensive and quantitative comparison is needed.

### The distribution of FSI events

An approach based on the critical point theory, and first used for combustion problems by Griffiths et al.^18^, has been utilised to identify the frequency and percentages of FSI events. In this approach, the reaction progress variable, defined in Eq. (1), is used to define critical points in the flame front. At a critical point within the flame (e.g. $x_i = a_i$), the gradient of the progress variable is zero. Thus, the Taylor series expansion around a critical point can be written as^18^:

4 $$\begin{aligned} c(a_i+x_i) = c(a_i) + \frac{x_j}{2} H_{ij}(c(a_i)) x_i + \dots \end{aligned}$$

where the Hessian $H_{ij}(c)$ describes, to second order accuracy, the local field. Provided that orientation is not important, the eigenvalues of $H_{ij}(c)$ fully define the local topological features to second order accuracy as well. This is the case, since the symmetric $H_{ij}(c)$ has real eigenvalues that give the curvature along each of the three orthogonal principal axes. To gain a more effective interpretation of the local topology, one can convert to spherical coordinates where the eigenvalues of $H_{ij}(c)$, $\lambda _1>\lambda _2>\lambda _3$, define the latitude $\varphi$ about the pole vector $[e_{\lambda _1},e_{\lambda _2},e_{\lambda _3}]$ and the longitude $\theta$ about the meridian vector $[e_{\lambda _1},0,-e_{\lambda _3}]$. The magnitude of the eigenvalues $\kappa =\sqrt{\lambda _1^2+\lambda _2^2+\lambda _3^2}$ gives an overall curvature measure, and can be treated as a scaling parameter. This leaves $\varphi$ and $\theta$ as shaping factors that, when normalised to lie between $-1/+1$, are given by^18^:

5 $$\begin{aligned} \begin{aligned} \theta&= \frac{6}{\pi } \arctan \left( \frac{(\lambda _1-2\lambda _2+\lambda _3)/\sqrt{6}}{(\lambda _1-\lambda _3)/\sqrt{2}} \right) \\ \varphi&= \frac{2}{\pi } \arctan \left( \frac{(\lambda _1+\lambda _2+\lambda _3)\cos (\theta \pi /6)/\sqrt{3}}{(\lambda _1-\lambda _3)/\sqrt{2}} \right) \end{aligned} \end{aligned}$$

**Figure 2:**
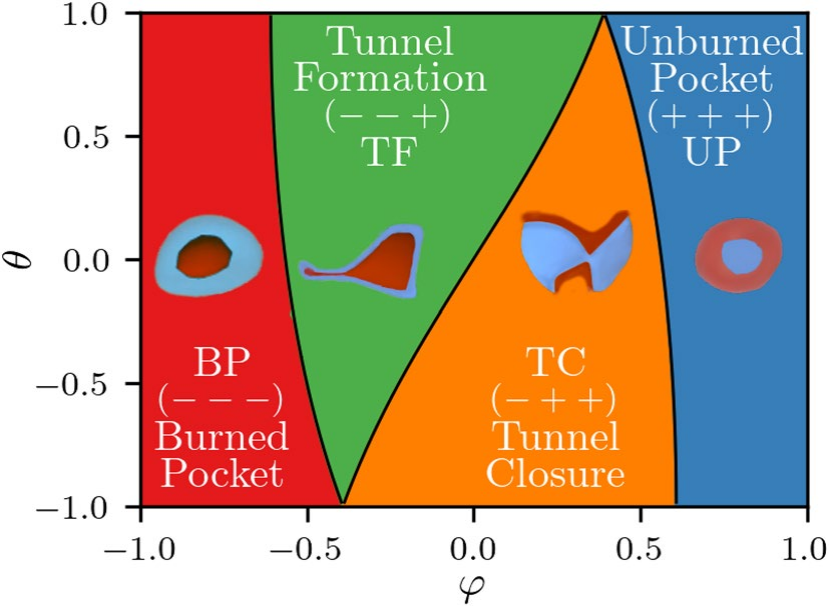
Visualisation of all feasible FSI topologies in the $\varphi$, $\theta$ domain.

Using the shape factors $\theta$ and $\varphi$, and based on the signs of the eigenvalues of $H_{ij}(c)$, all the feasible local topologies of FSI can be identified. The set of all possible topologies in the ($\varphi$, $\theta$) space is shown in Fig. 2.

Four types of FSI topologies can be identified in Fig. 2. These are: Burned gas Pocket (BP), Tunnel Formation (TF), Tunnel Closure (TC) and Unburned gas Pocket (UP) topologies, corresponding to $H_{ij}(c)$ eigenvalues with signs ($---$), ($--+$), ($-++$) and ($+++$), respectively. The pocket topologies represent spherical flames propagating outwards (BP) or inwards (UP), while the tunnel topologies represent cylindrical flames propagating towards (TC) or away from (TF) a common axis.

In the present study, the critical points associated with the FSI events are identified based on the methodology adopted by Griffiths et al.^18^

**Figure 3:**
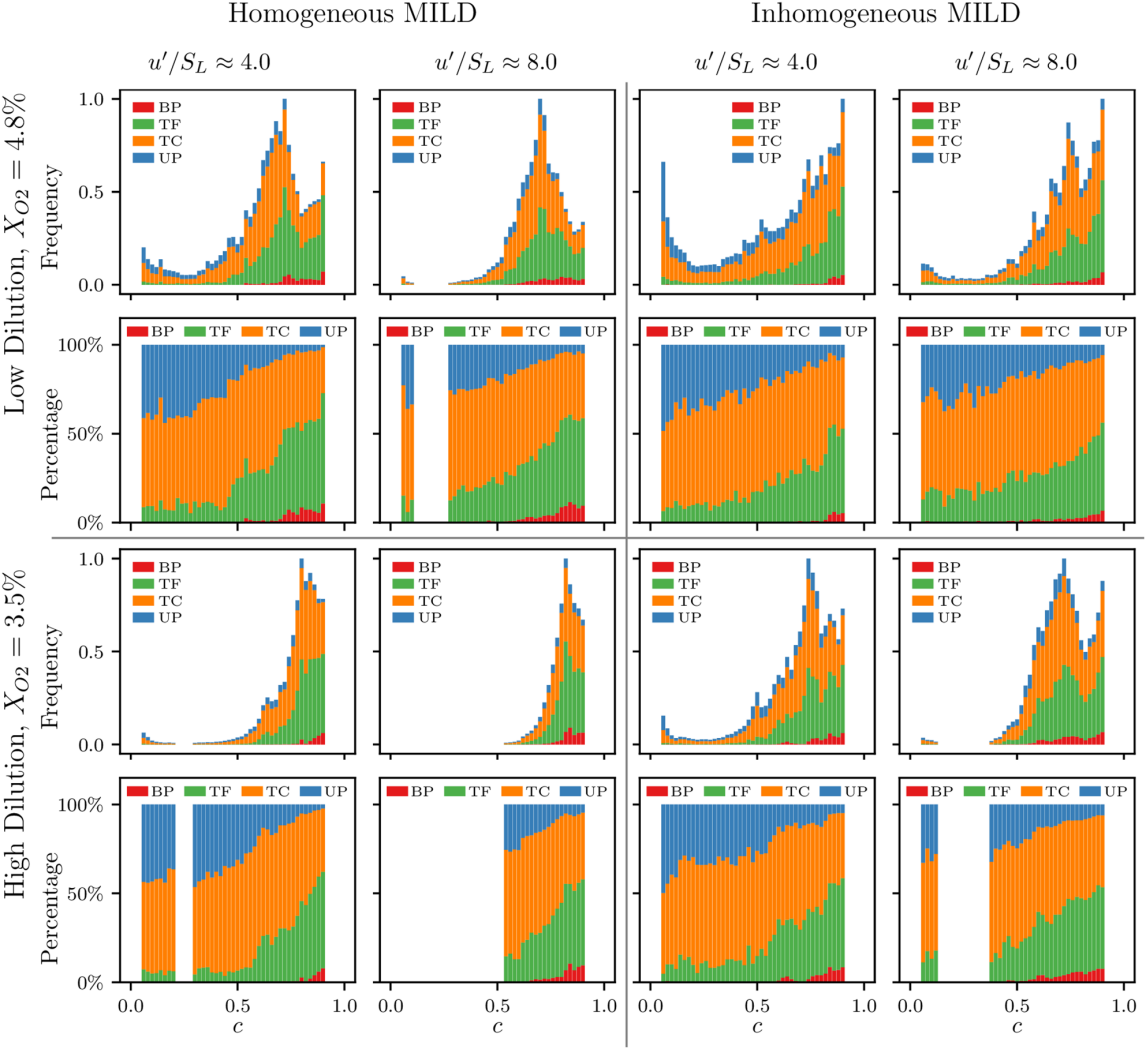
The distribution of the four FSI topologies, and their percentages, in the region $0.05 \le c \le 0.95$ for all cases considered. The topology count is normalised by the value of the maximum frequency in each case.

Figure 3 shows the occurrence frequency of different FSI topologies, and their percentages, at different *c* values in the region $0.05 \le c \le 0.95$ for all cases considered. The frequency counts shown in Fig. 3 are normalised by the value of the maximum frequency in each case.

It can be seen from Fig. 3 that both the homogeneous and inhomogeneous cases exhibit a significant amount of FSI events within the flame front for both dilution levels. In all cases, the majority of FSI events occur at $c > 0.5$, with the peak FSI frequencies occurring between $c = 0.6$ and $c=0.75$ in most cases. This aligns with the results reported by Abo-Amsha and Chakraborty^19^ for n-heptane MILD combustion where the peak frequency of FSI events was also found to be within the flame front ($c \approx 0.5$). A notable exception to this trend is the inhomogeneous mixture case at low dilution where the peak frequency of FSI events is clearly shifted further towards the burned gas side. A possible explanation for this behaviour is that the combination of mixture inhomogeneity, which introduces increased levels of reactivity^23,24^, and higher oxygen concentration leads to this shift in peak FSI frequency to higher *c* values. However, it remains difficult to categorically establish the exact reason for, or the mechanism behind, this behaviour. Moreover, due to the high computational cost of DNS, only a limited number of cases could be simulated which precluded the identification of any meaningful trends.

From Fig. 3 one can see that the distribution of FSI events in *c* space under MILD combustion conditions is qualitatively different from that found in turbulent premixed combustion, where two peak frequencies of FSI events occur at $c \approx 0$ and $c \approx 0.9$^18,25^. Figure 3 also shows that, in all cases, the cylindrical topologies have the highest percentage share (60%–80%) across the flame front with higher likelihood of TC topologies towards the unburned gas side, while TF type events occur more often towards the burned gas side. On the other hand, the pocket topologies are mostly clustered towards the burned (for BP) and unburned gas (for UP) sides and constitute between 15 and 40% of all FSI events depending on *c* value.

The total number of FSI events are shown in Table 3. This provides a quantitative overview of the effect of turbulence intensity, dilution level and mixture inhomogeneity on the occurrence of FSI events. Here, a quantitative comparison between cases is possible since the analysis has been carried out on the same number of snapshots (50 snapshots per case, with the same time interval between snapshots) for all cases. Table 3 shows that the total count of FSI events increases with increasing turbulence intensity (about two to five folds increase) and dilution levels (three to five folds increase). This could be due to the fact that the increase in initial turbulence intensity increases the wrinkling of the flame surface which in turn enhances the likelihood of FSI events. The increase in dilution level leads to the flame filling a larger percentage of the domain as can be seen from Fig. 1. This provides more opportunity for flame surfaces to interact leading to increased frequency of FSI events. It can also be seen from Table 3 that mixture inhomogeneity acts to reduce the frequency of FSI events for both dilution levels and turbulence intensities. This can be attributed to an increase in the progress variable reaction rate due to mixture inhomogeneity^23,24^, which results in the combustion process being completed in smaller portion of the domain. Thus, the region $0.05 \le c \le 0.95$ fills up a smaller portion of the domain which reduces the opportunity for FSI events to occur. This behaviour can be visually supported in Fig. 1.

**Table 3:** The total number of FSI events in all cases considered.

	Homogeneous MILD		Inhomogeneous MILD	
	$u'/S_L= 4.0$	$u'/S_L= 8.0$	$u'/S_L \approx 4.0$	$u'/S_L \approx 8.0$
LD ($X_{O2}=4.8\%$)	$2.23 \times 10^5$	$8.58 \times 10^5$	$1.10 \times 10^5$	$2.67 \times 10^5$
HD ($X_{O2}=3.5\%$)	$7.28 \times 10^5$	$4.04 \times 10^6$	$3.28 \times 10^5$	$1.02 \times 10^6$

### FSI topologies in the $\kappa _m-\kappa _g$ plane

**Figure 4:**
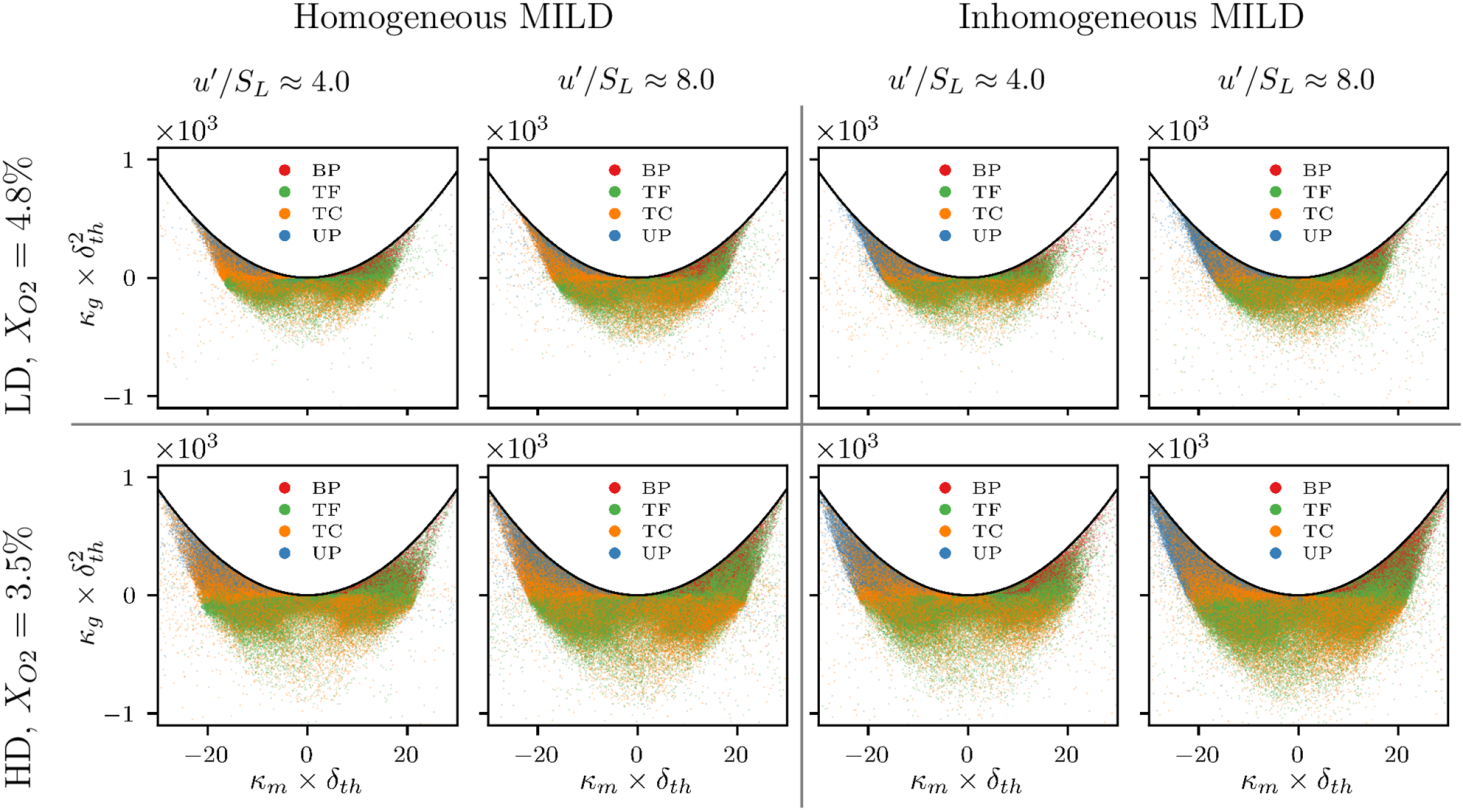
Scatter of $\kappa _g \times \delta _{th}^2$ with $\kappa _m \times \delta _{th}$ for the FSI events in the region $0.05 \le c \le 0.95$ for all cases considered. Scatter is coloured by the different FSI topologies.

To gain further insights into the topology of the FSI events present in MILD combustion, an alternative approach for analysing the flame surface geometry can be utilised. Following Dopazo et al.^26^, the local flame surface geometry can be characterised in terms of the local mean curvature ($\kappa _m = 0.5(\kappa _1 + \kappa _2) = 0.5 \partial n_i/\partial x_i$), and the Gaussian curvature ($\kappa _g = \kappa _1 \times \kappa _2$), where $\kappa _1$, $\kappa _2$ are the principal curvatures and $\textbf{n}=-\nabla c/|\nabla c|$ is the flame normal vector. In the ($\kappa _m$, $\kappa _g$) plane, points in the region where $\kappa _g > \kappa _m^2$ imply complex principal curvatures which is unphysical. Elsewhere on the ($\kappa _m$, $\kappa _g$) plane, a positive value for $\kappa _g$ indicates elliptic, cup-like topology, whereas a negative $\kappa _g$ implies a hyperbolic, saddle-like geometry. Here, a positive (negative) value for $\kappa _m$ indicates that the flame surface is convex (concave) towards the reactants’ side. Figure 4 shows the scatter of FSI events in the ($\kappa _m$, $\kappa _g$) space for all cases considered. It can be seen from Fig. 4 that the inward propagating pocket topologies (UP) are clustered in the region of elliptic geometry with negative mean curvature (i.e. $\kappa _g > 0$ and $\kappa _m < 0$), while the outward propagating pocket topologies (BP) are associated with positive mean curvature, elliptic topology (i.e. $\kappa _g > 0$ and $\kappa _m > 0$). Figure 4 also shows that TF topologies are mainly related to elliptic geometries with positive mean curvature and hyperbolic saddle geometries with negative mean curvature. On the other hand, the TC topologies show the opposite trend and are closely associated with hyperbolic saddle geometries with positive mean curvature and elliptic geometries with negative mean curvature. Both turbulence intensity and dilution level do not significantly affect the distributions of the FSI topologies in the ($\kappa _m$, $\kappa _g$) plane, and the distribution remains qualitatively similar for both homogeneous and inhomogeneous MILD combustion.

### The evolution of $\sigma \equiv | \nabla c |$ near FSI events

Since the critical points are located where $|\nabla c |= 0$, it is worth investigating the evolution of the surface density function (i.e. $\sigma \equiv | \nabla c |$) in the vicinity of critical points. For this purpose, it is useful to consider the transport equation of $\sigma$^16,17,27^:

6 $$\begin{aligned} \frac{\partial \sigma }{\partial t} + u_j \frac{\partial \sigma }{\partial x_j} = -\underbrace{n_i n_j \frac{\partial u_i}{\partial x_j}\sigma }_{\text {Production}} \, -\underbrace{n_j \frac{\partial }{\partial x_j}\left( \frac{A_\xi }{\rho } \right) }_{\text {inhomogeneity term}} -\underbrace{n_j \frac{\partial }{\partial x_j} \left\{ \frac{1}{\rho } \left[ {\dot{\omega }} - n_i \frac{\partial }{\partial x_i} (\rho D \sigma ) \right] \right\} }_{\text {Kinematic Restoration (KR)}} +\underbrace{n_j \frac{\partial }{\partial x_j} (2 D \kappa _m \sigma )}_{\text {Area Dissipation (AD)}} \end{aligned}$$

where $\rho$ is the density of the reactant’s mixture, while ${\dot{\omega }}$ and *D* are the reaction rate and diffusivity of the reaction progress variable, respectively. In Eq. (6) the first term on the right hand side represents the production of $\sigma$ due to straining by the fluid flow^16^, while the second term accounts for the effect of mixture inhomogeneity on the evolution of $\sigma$ with $A_\xi$ defined as^28^:

7 $$\begin{aligned} A_\xi = \left\{ \begin{array}{ll} \dfrac{-2 \rho D \sigma }{\xi } \, n_j \dfrac{\partial \xi }{\partial x_j} &{}\text {for} \quad \xi \le \xi _{st}\\ \dfrac{2 \rho D \sigma }{1-\xi } \, n_j \dfrac{\partial \xi }{\partial x_j} &{}\text {for} \quad \xi > \xi _{st} \end{array} \right. \end{aligned}$$

The last two terms in Eq. (6) are named the kinematic restoration (KR) and dissipation (AD) terms, respectively^16,29^. The kinematic restoration term arises from the flame normal propagation due to the reaction and flame normal diffusion effects, while the dissipation term represents the dissipation of the flame surface area due to diffusive effects^16^.

Kollmann and Chen^16^ showed, based on a 2D theoretical analysis of conventional premixed flames, that the kinematic restoration (KR) and area dissipation (AD) terms contain unbounded contributions in the vicinity of critical points representing cylindrical FSI events (i.e. TC and TF topologies). These contributions behave as 1/*r* as $r \rightarrow 0$, where *r* is the distance from the critical point. However, Kollmann and Chen^16^ reported that the singular contribution arising in the dissipation term exactly cancels out the ones present in the kinematic restoration term^16^. Thus, the $\sigma$ balance equation remains bounded in the neighbourhood of cylindrical FSI events. Moreover, Trivedi et al.^27^ reported, based on theoretical analysis and DNS data, that the findings of Kollmann and Chen^16^ remain valid for 3D conventional turbulent premixed flames. Trivedi et al.^27^ also showed that the instantaneous profiles of the kinematic restoration (KR) and area dissipation (AD) terms attain similar magnitudes but opposite signs in the vicinity of TC- and TF-type critical points, such that the terms would cancel out on average. However, a similar analysis is yet to be conducted under MILD combustion conditions. It is worthwhile to assess the behaviour of the kinematic restoration (KR) and dissipation (AD) terms at the cylindrical FSI events in MILD combustion due to the significantly different physical processes involved in MILD combustion compared to conventional premixed flames^11^, Moreover, unlike in the analyses by Kollmann and Chen^16^ and Trivedi et al.^27^, the current study focuses on the behaviour of the KR and AD terms conditionally averaged upon the reaction progress variable in the vicinity of cylindrical critical points.

**Figure 5:**
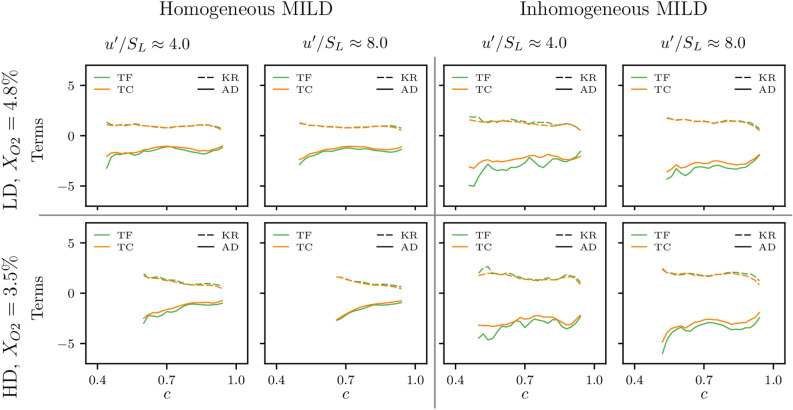
Profiles of the mean values of the kinematic restoration (KR) and area dissipation (AD) terms conditioned upon *c* for the two cylindrical topologies (TF and TC) in all the cases considered. The terms are normalised by $\delta _{th}^2/S_L$.

The mean profiles of the kinematic restoration (KR) and area dissipation (AD) terms conditioned upon *c* in the vicinity of the two cylindrical FSI topologies (TF and TC) are shown in Fig. 5. The terms in Fig. 5 are normalised by $\delta _{th}^2/S_L$ and plotted only for *c* values where significant occurrences of the cylindrical FSI events (i.e. TF and TC) are present. These *c* locations can be seen from Fig. 3.

Figure 5 shows that the mean profiles of kinematic restoration and dissipation terms conditioned upon *c* attain similar magnitude, but opposite signs in the neighbourhood of the cylindrical FSI topologies. This, in turn, suggests that there is a significant likelihood for both terms to cancel each other in the vicinity of the TF- and TC-type critical point, which is consistent with the observations made in previous DNS studies for premixed turbulent flames^27^. However, an exact cancellation for kinematic restoration and dissipation terms should not be expected since both terms are evaluated near the critical point and exact cancellation is only expected to occur at the critical point. It is also evident from Fig. 5 that the approximate cancellation of the kinematic restoration and dissipation contributions occurs regardless of the turbulence intensity, dilution level and mixture inhomogeneity. Figure 5 also reveals that the kinematic restoration and dissipation terms show little change in behaviour within the parameter ranges considered in this study. This, in turn, confirms that the analytical conclusion of Kollmann and Chen^16^, where the singular contributions in KR and AD obtained at TF- and TC-type critical points cancel each other, is valid and the overall balance equation for $\sigma$ remains bounded in the neighbourhood of cylindrical FSI events in MILD combustion. Thus, there is no need for a separate model treatment for singularities in $\sigma \equiv | \nabla c |$ based quantities (e.g. scalar dissipation rate $N_c = D | \nabla c |^2$) due to FSI in MILD combustion.

### FSI topologies in the reaction dominated and flame propagation regions

Since MILD combustion contains an interplay between ignition and localised extinction events where auto-igniting kernels grow as they are convected in the fluid and lead to propagation-like behaviour^13^, a further insight into the topological features of flame surfaces in MILD combustion can be attained by analysing the FSI events in the two regions: (1) where chemical reaction is dominant, and (2) where propagation-like behaviour is more prevalent. Minamoto et al.^9^ have shown that, by balancing the convection-diffusion and reaction contributions in the transport equation of the reaction progress variable, the reaction dominated (RD) and the propagating flames (PF) regions can be isolated. Starting from the transport equation of the reaction progress variable, which can be written as:

8 $$\begin{aligned} \frac{\partial \rho c}{\partial t} + \underbrace{\frac{\partial \rho u_i c}{\partial x_i}}_{{\mathscr {C}}: \, \textrm{convection}} = \underbrace{\frac{\partial }{\partial x_j} \left( \rho D \frac{\partial c}{\partial x_j} \right) }_{{\mathscr {D}}: \, \textrm{diffustion}} + \underbrace{{\dot{\omega }}}_{{\mathscr {R}}: \, \textrm{reaction}}+ \underbrace{A_{\xi }^c}_{\text {inhomogeneity term}}, \end{aligned}$$

one can define a factor ${\mathscr {B}}$ as follows:

9 $$\begin{aligned} {\mathscr {B}} \equiv | {\mathscr {C}} - {\mathscr {D}} | - {\mathscr {R}}, \end{aligned}$$

where ${\mathscr {C}}$, ${\mathscr {D}}$ and ${\mathscr {R}}$ are the convection, diffusion and reaction terms in the reaction progress variable transport equation and are shown in Eq. 8. It has been shown elsewhere^23,28^ that $A_\xi ^c$ assumes smaller magnitudes than ${\mathscr {R}}$ and ${\mathscr {D}}$ in inhomogeneous mixture combustion and the mixture inhomogeneity effects on the reaction progress variable transport are principally felt through ${\mathscr {R}}$ and ${\mathscr {D}}$. Thus, the value of ${\mathscr {B}}$ can be used to characterise reaction dominated and propagating flame regions, such that the regions where ${\mathscr {B}} > 0$ are termed the propagating flames (PF) regions, while the reaction dominated (RD) regions are identified where ${\mathscr {B}} < 0$.

**Figure 6:**
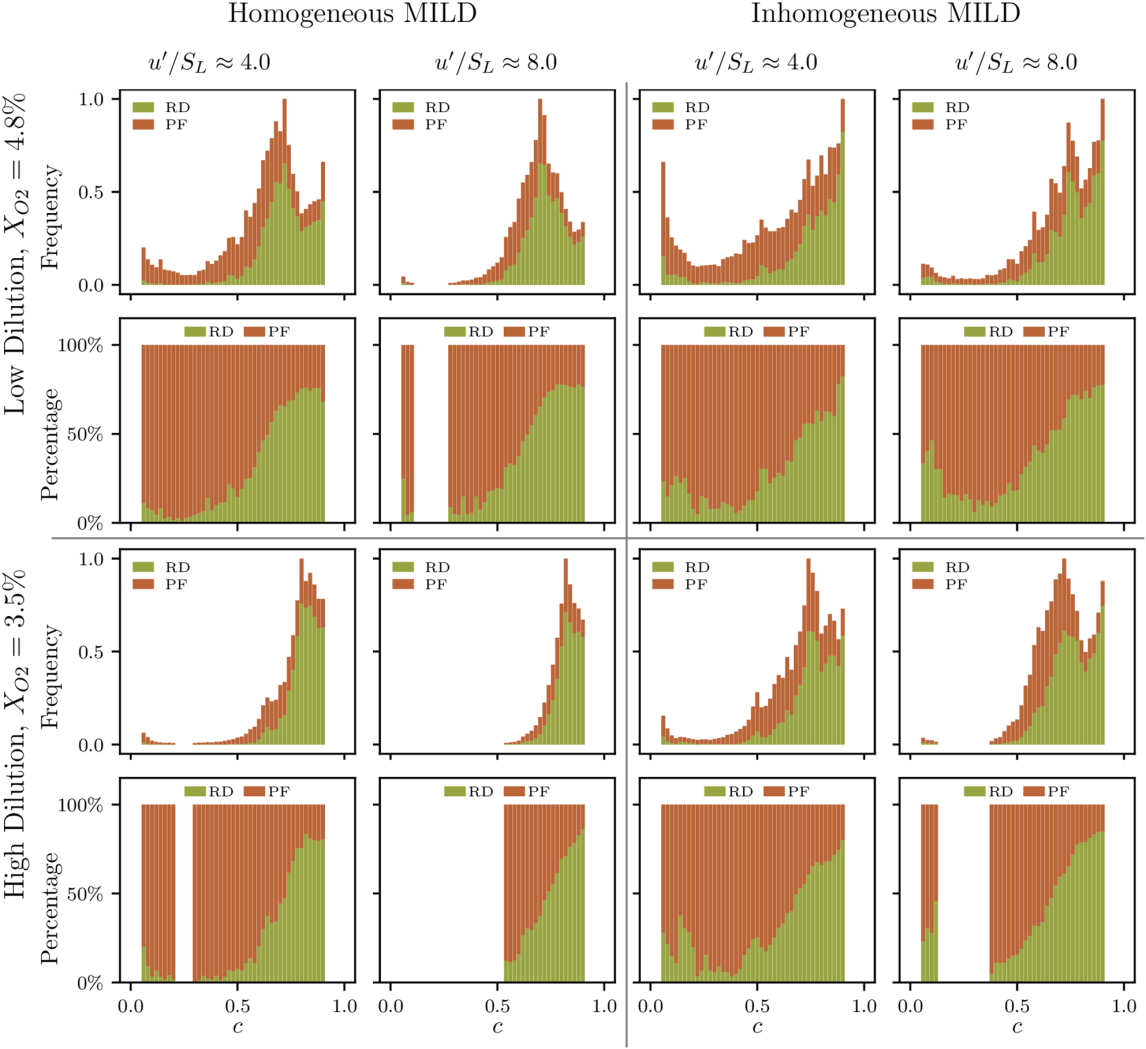
The frequency count and the percentages of total FSI events in the reaction dominated (RD) and propagating flame (PF) regions for $0.05 \le c \le 0.95$ in all cases considered. The frequency count is normalised by the value of the maximum frequency in each case.

Figure 6 shows the frequency count, and the percentages, of all FSI events occurring in the reaction dominated and propagating flame regions. Minamoto et al.^9^ have suggested that the majority of flame-flame interactions would occur in the reaction dominated region. However, Fig. 6 shows that the majority of FSI events present towards the unburned gas side occur in the propagating flame region, which contradicts the suggestion of Minamoto et al.^9^ However, this contradiction is balanced by the relatively small number of FSI events occurring in the unburned gas side compared to those present towards the burned gas side where maximum frequency of FSI events are found and the majority of these events happen in the reaction dominated region as suggested by Minamoto et al.^9^

**Figure 7:**
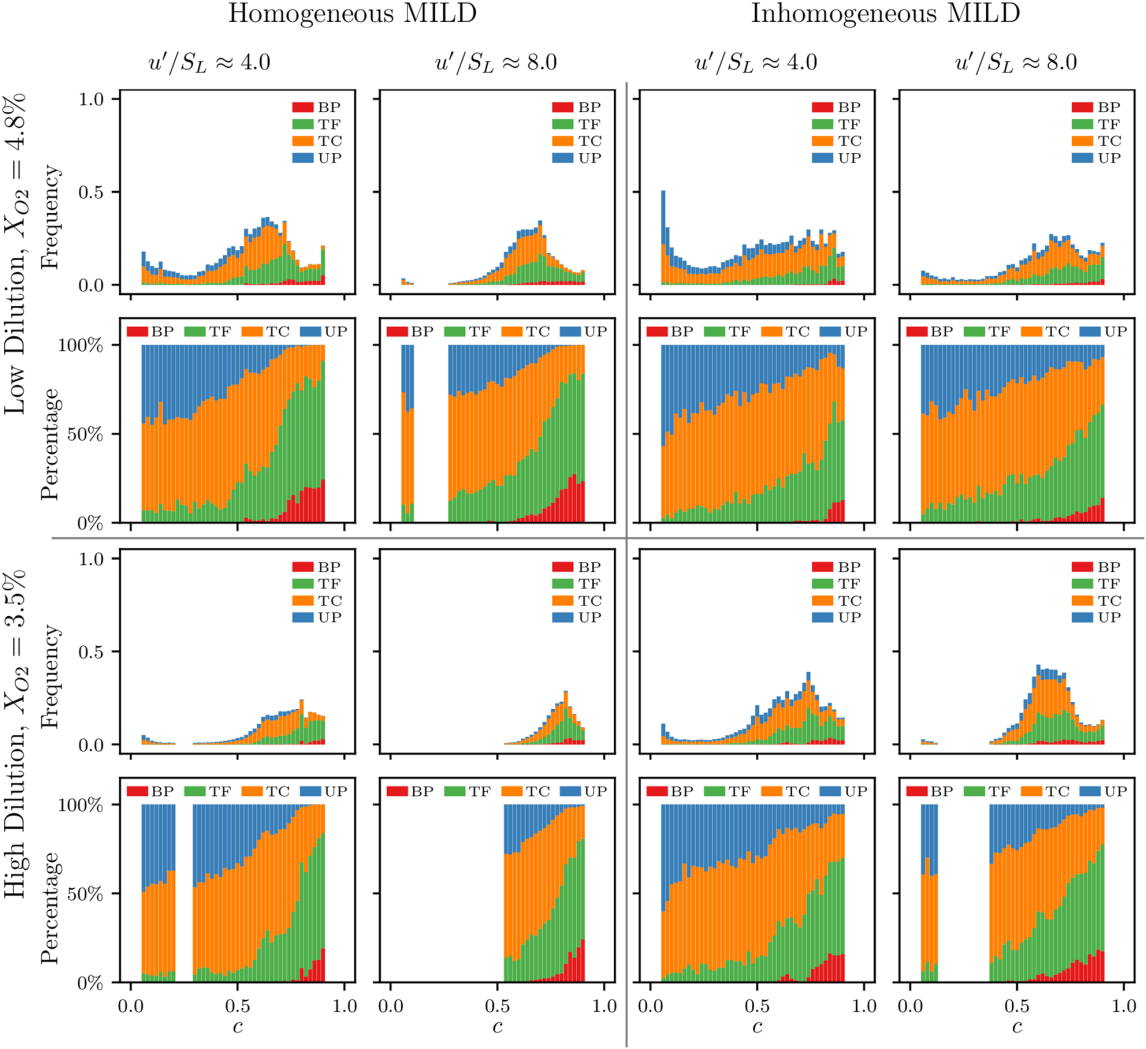
The distribution and percentages of the four FSI topologies occurring in the propagating flame (PF) region for $0.05 \le c \le 0.95$ in all cases considered. The topology count is normalised by the value of the maximum frequency in each case.

**Figure 8:**
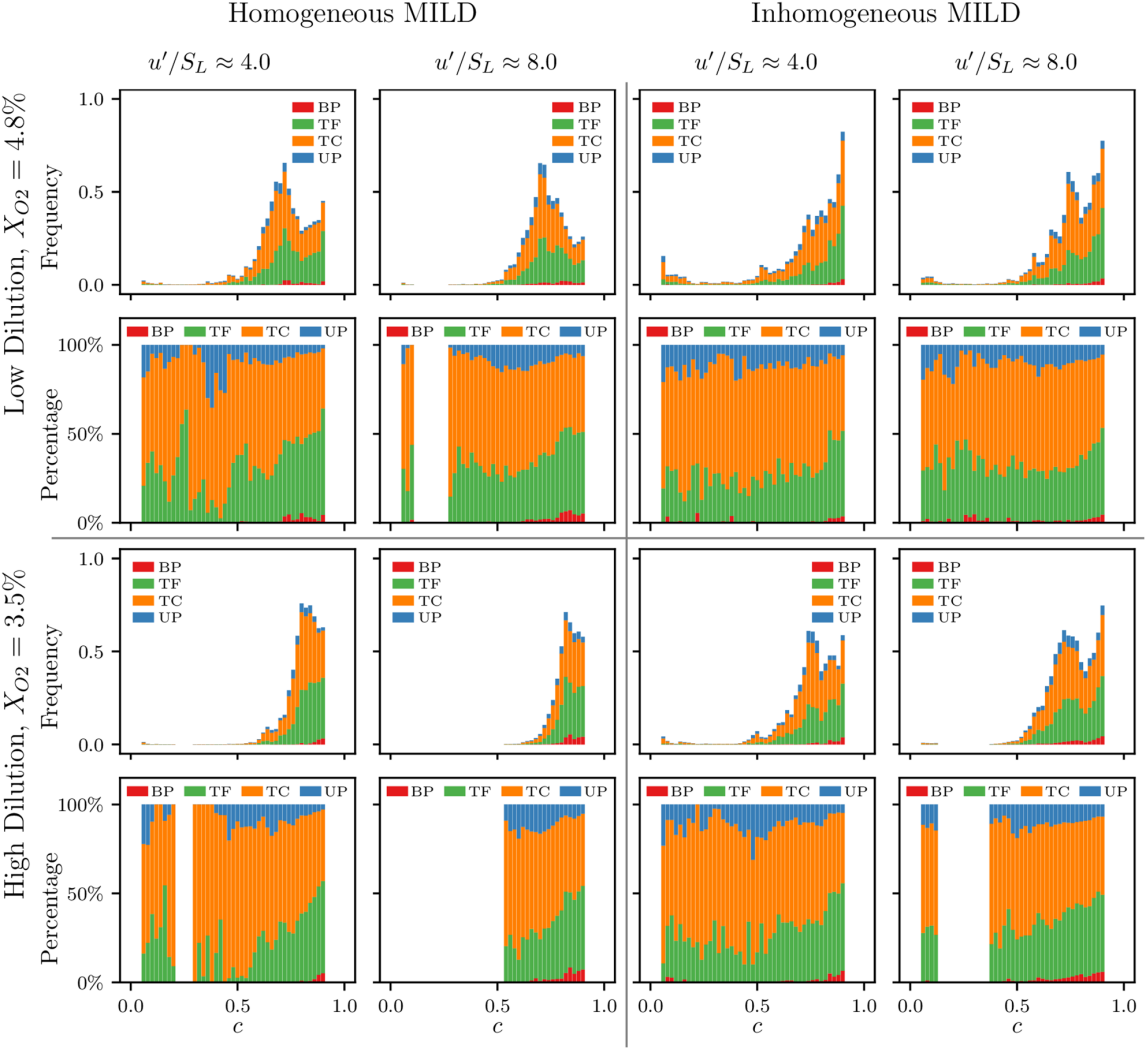
The distribution and percentages of the four FSI topologies occurring in the reaction dominated (RD) region for $0.05 \le c \le 0.95$ in all cases considered. The topology count is normalised by the value of the maximum frequency in each case.

Figures 7 and 8 show the distribution of occurrence frequency and the percentages of the four FSI topologies across the $0.05 \le c \le 0.95$ range in the propagating flame and reaction dominated regions, respectively, in all cases considered. It can be seen from Figs. 7 and 8 that a similar trend to that seen in Fig. 3 is maintained whereby the location of maximum frequency of FSI events remains towards the burned gas side at $c>0.6$. Figure 7 also shows that, similar to Fig. 3, the cylindrical FSI events remain the most likely in the propagating flame region with TC topologies being more frequent towards the unburned gas side, and TF having the highest percentage towards the burned gas side. Comparing Fig. 7 with Fig. 8 shows that a large percentage of pocket topologies occur in the propagating flame region where UP has a higher percentage share towards the unburned gas side, and BP topologies are only present towards the burned gas side. On the other hand, Fig. 8 shows that the vast majority of FSI events in the reaction dominated region are cylindrical in type (i.e. TF and TC) and relatively few pocket topologies are present (i.e. only a small percentage of UP topologies with very little presence for BP topologies). Moreover, it can be seen from Fig. 8 that, unlike the trend shown in Fig. 3, the percentage of TC and TF topologies remain almost constant throughout the analysed range of *c* values.

In summary, the change in turbulence intensity and dilution level has been shown to influence the total amount of FSI events, but the qualitative shape of FSI topologies distribution in *c* space remained the same for the range of turbulence intensities and dilution levels considered here. It has also been found that the introduction of mixture inhomogeneity reduces the overall frequency of FSI events. Thus, the effects of turbulence intensity, dilution level and mixture inhomogeneity need to be taken into consideration in the attempts to extend flame surface based models to MILD combustion.

## Summary and conclusions

The frequency of FSI events and the distribution of the topologies associated with it have been investigated in $\mathrm {CH_4}$-based homogeneous and inhomogeneous mixture EGR-type MILD combustion under different values of $\mathrm {O_2}$ dilution (i.e. $X_{O2} = 4.8\%$ and $3.5\%$) and turbulence intensities using three-dimensional DNS with a skeletal chemical mechanism. The findings of the current analysis can be summarised as follows:

- Both homogeneous and inhomogeneous mixture MILD combustion exhibit significant amounts of FSI events and the frequency of occurrence of FSI events peaks towards the burned gas side at $c = 0.6-0.75$.
- The frequency of FSI events increases between three to five folds with increasing dilution level and turbulence intensity, whereas mixture inhomogeneity acts to reduce the frequency of FSI events by around 50%.
- In all cases, the FSI topologies associated with tunnel formation and tunnel closures are more frequent (accounting for 60%–85% of all FSI events) than spherical topologies (i.e. unburned gas and burned gas pockets account for 15%–40%).
- There is a high probability of finding burned gas (unburned gas) pockets near the burned (unburned) gas side, but these events become rare towards the unburned (burned) gas side in all cases considered here.
- When analysing the FSI events in the ($\kappa _m$, $\kappa _g$) plane, it has been found that the inward propagating pocket topologies (UP) are clustered in the region of elliptic geometry with negative mean curvature, while the outward propagating pocket topologies (BP) are associated with positive mean curvature, elliptic topology. In contrast, the outward propagating tunnel topology (TF) is related to elliptic geometries with positive mean curvature or hyperbolic saddle geometries with negative mean curvature, while the inward propagating tunnel topology (TC) is associated with hyperbolic saddle geometries with positive mean curvature or elliptic geometries with negative mean curvature.
- The kinematic restoration (KR) and dissipation (AD) terms in the $\sigma \equiv | \nabla c |$ transport equation tend to cancel each other in the vicinity of the cylindrical topologies (i.e. TC and TF). Therefore, the singular contributions present in the (KR) and (AD) terms in the vicinity of TC and TF topologies do not affect the overall balance equation of $\sigma$ which remains bounded. Hence, the presence of large frequency of FSI events in MILD combustion may not affect the closure of the quantities dependent on $| \nabla c |$ (e.g. scalar dissipation rate $N_c=D|\nabla c|^2$).
- The topologies of FSI events in both the reaction dominated and propagating flame regions have been analysed. It has been found that, towards the burned gas side, a big portion (up to 75%) of FSI events occurs in the reaction dominated region, while most ($\approx$ 95%) of FSI events present towards the unburned gas side occur in the propagating flame region. Moreover, while the cylindrical FSI topologies remained the most frequent in both the reaction dominated ($\approx$ 90%) and propagating flame (60%–90%) regions, the propagating flame region had a larger share (20%–40%) of pocket topologies compared to that present in the reaction dominated region (5%–10%).

In summary, the findings of this study show that turbulence intensity, dilution level and mixture inhomogeneity all influence the total amount of FSI events, and thus need to be taken into consideration when using flame surface based-modelling approaches.

## Methods

The 3D DNS data for the current analysis is generated using a well-known compressible DNS code SENGA+^30^ which solves the standard conservation equations of mass, momentum, energy and species for turbulent reacting flows. The spatial differentiation operations are approximated using a tenth order central difference scheme for the internal grid points, but the order of accuracy reduces gradually to a one-sided fourth-order scheme at the non-periodic boundaries. A fourth-order, low-storage Runge-Kutta scheme with adaptive time step control is used for explicit time advancement. The error threshold for the time step adaption was set at $10^{-4}$. For all cases, a cubic domain with an edge size $L=10 \, \text {(mm)}$, discretised by a uniform Cartesian grid of $252^3$, has been used. The grid spacing ensures that the thermal flame thickness $\delta _{th}$ is resolved by using 12 grid points and the Kolmogorov length scale ($\eta$) is resolved by at least 1.5 grid points for all turbulence levels. The boundary conditions are specified according to the Navier–Stokes Characteristic Boundary Condition (NSCBC) approach^31^. An inflow boundary condition with specified density was assigned on the left-hand side boundary in the *x*-direction, while a partially non-reflective outflow boundary condition was specified for the right-hand side boundary in the *x*-direction. All other boundaries of the domain were taken to be periodic.

The MILD combustion simulations have been conducted in two phases. The first phase aims to mimic the mixing of combustion products with fresh reactants that occurs in an Exhaust Gas Recirculation (EGR)-type combustor, while the second phase simulates the combustion process. The initial scalar fields in phase 1 are prepared according to the methodology reported by Minamoto et al.^8^ for homogeneous mixture MILD combustion, and by Doan et al.^12^ for inhomogeneous mixture MILD combustion. Only a brief description of these methodologies is provided here for the sake of brevity. Interested readers are referred to Refs.^8,12^ for more details. The procedure for the generation of the initial scalar fields is summarised as follows:

1. A homogeneous isotropic turbulence field generated using a pseudo-spectral method^32^ following the Batchelor-Townsend spectrum^33^, is used to initialise the turbulent velocity fluctuations. Table 2 shows the turbulence intensities and turbulent length scales in the current simulations.
2. For the homogeneous mixture combustion cases, a 1D freely-propagating laminar premixed flame at $\phi =0.8$ is simulated with each oxidiser stream from Table 1. For the inhomogeneous mixture combustion cases, a set of 1D freely-propagating laminar premixed flames, with each oxidiser stream from Table 1, is simulated for a range of equivalence ratios, namely $\phi = 0.3-1.3$. Each oxidiser stream in Table 1 correspond to a dilution level.
3. For homogeneous mixture combustion simulations, an initial 3D *c* field with a bimodal distribution of *c* is generated for a mean reaction progress variable of 0.5 and a prescribed integral length scale $\ell _c=1.5 \, (mm)$. In the case of inhomogeneous mixture combustion simulations, two independent 3D fields with bimodal distributions of *c* and $\phi$ are generated for $\langle c \rangle =0.5$ and $\langle \phi \rangle =0.8$ with integral length scales of $\ell _c=1.5 \, (mm)$ and $\ell _\phi =2.2 \, (mm)$, respectively. The peaks of the $\phi$ bimodal distribution are at $\phi = 0.3$ and $\phi = 1.3$. The 3D fields with bimodal distributions of *c* and $\phi$ were generated following the method developed by Eswaran and Pope^34^.
4. The species mass fractions from the 1D premixed flame solutions are parameterised as functions of the progress variable *c* for the homogeneous mixture cases and both *c* and $\phi$ for the inhomogeneous mixture cases. These functions are then used to populate the bimodal fields from the previous step and create three dimensional scalar fields.
5. The scalar fields from Step 4 are allowed to interact with the turbulence field (generated in Step 1) without chemical reaction for about one turnover time (i.e. $t_e=\ell /u'$) in a periodic domain representing the EGR process in MILD combustion.

At the end of the last step, the mean and variance of the pre-processed *c* fields are found to be $\langle c \rangle \approx 0.5$ and $\langle c'^2 \rangle \approx 0.09$, whereas the mean and variance of the pre-processed $\phi$ fields in the inhomogeneous mixture combustion cases are found to be $\langle \phi \rangle \approx 0.8$ and $\langle \phi '^2 \rangle \approx 0.16$, respectively.

The scalar fields resulting from the first phase act as both an initial condition and inflow fields for the second phase, where these scalar fields are fed into the simulation domain with a mean inlet velocity $U_{in} = 20 \, (m/s)$. The simulations were conducted for 2.5 flow through times (i.e. 2.5$\tau$, where $\tau = L/U_{in}$). The snapshots used for this analysis have been chosen between 1.5 and 2.5 flow through times in steps of $\tau /50$. This allowed the effects of the initial conditions to leave the domain. It has been ensured that the statistics presented in this analysis do not change significantly if half of the snapshots between 1.5 and 2.5 flow through times were used instead of all snapshots. This ensures that a satisfactory level of statistical convergence has been achieved. The above numerical procedure has been extensively used in the literature for the study of turbulent MILD combustion^7–12,19,23,35^

## Data Availability

The datasets used and/or analysed during the current study are available from the corresponding author on reasonable request.

## References

[1] Wünning J, Wünning J (1997). Flameless oxidation to reduce thermal no-formation. Prog. Energy Combust. Sci..

[2] Katsuki M, Hasegawa T (1998). The science and technology of combustion in highly preheated air. Symp. (Int.) Combust..

[3] Cavaliere A, de Joannon M (2004). Mild Combustion. Prog. Energy Combust. Sci..

[4] Plessing T, Peters N, Wünning J (1998). Laseroptical investigation of highly preheated combustion with strong exhaust gas recirculation. Symp. (Int.) Combust..

[5] Özdemir I, Peters N (2001). Characteristics of the reaction zone in a combustor operating at mild combustion. Exp. Fluids.

[6] Dally B, Riesmeier E, Peters N (2004). Effect of fuel mixture on moderate and intense low oxygen dilution combustion. Combust. Flame.

[7] Minamoto Y, Swaminathan N, Cant R, Leung T (2014). Reaction zones and their structure in MILD combustion. Combust. Sci. Technol..

[8] Minamoto Y, Dunstan TD, Swaminathan N, Cant R (2013). DNS of EGR-type turbulent flame in MILD condition. Proc. Combust. Inst..

[9] Minamoto Y, Swaminathan N, Cant S, Leung T (2014). Morphological and statistical features of reaction zones in MILD and premixed combustion. Combust. Flame.

[10] Minamoto Y, Swaminathan N (2014). Scalar gradient Behaviour in MILD combustion. Combust. Flame.

[11] Awad H, Abo-Amsha K, Ahmed U, Chakraborty N (2021). Comparison of the reactive scalar gradient evolution between homogeneous MILD combustion and premixed turbulent flames. Energies.

[12] Doan N, Swaminathan N, Minamoto Y (2018). DNS of MILD combustion with mixture fraction variations. Combust. Flame.

[13] Doan N, Swaminathan N (2019). Autoignition and flame propagation in non-premixed MILD combustion. Combust. Flame.

[14] Dunstan TD, Swaminathan N, Bray KNC, Kingsbury NG (2013). Flame Interactions in Turbulent Premixed Twin V-Flames. Combust. Sci. Technol..

[15] Minkowski H (1910). Die Grundgleichungen für die elektromagnetischen Vorgänge in bewegten Körpern. Math. Ann..

[16] Kollmann W, Chen J (1998). Pocket formation and the flame surface density equation. Symp. (Int.) Combust..

[17] Chen J, Echekki T, Kollmann W (1999). The mechanism of two-dimensional pocket formation in lean premixed methane-air flames with implications to turbulent combustion. Combust. Flame.

[18] Griffiths R, Chen J, Kolla H, Cant R, Kollmann W (2015). Three-dimensional topology of turbulent premixed flame interaction. Proc. Combust. Inst..

[19] Abo-Amsha K, Chakraborty N (2023). Flame self-interaction and flow topology in turbulent homogeneous mixture n-Heptane MILD combustion. Flow Turbul. Combust..

[20] Smooke M, Giovangigli V, Smooke M (1991). Formulation of the premixed and nonpremixed test problems. Reduced Kinetic Mechanisms and Asymptotic Approximations for Methane-Air Flames: A Topical Volume.

[21] Hélie J, Trouvé A (1998). Turbulent flame propagation in partially premixed combustion. Symp. (Int.) Combust..

[22] Bilger R, Libby P, Williams F (1980). Turbulent flows with nonpremixed reactants. Turbulent Reacting Flows, Topics in Applied Physics.

[23] Abo-Amsha K, Chakraborty N (2023). Surface density function and its evolution in homogeneous and inhomogeneous mixture n-heptane MILD combustion. Combust. Sci. Technol..

[24] Awad H, Abo-Amsha K, Ahmed U, Swaminathan N, Chakraborty N (2023). A priori direct numerical simulation assessment of mild combustion modelling in the context of Reynolds averaged Navier–Stokes simulations. Flow Turbul. Combust..

[25] Trivedi S, Nivarti G, Cant R (2019). Flame self-interactions with increasing turbulence intensity. Proc. Combust. Inst..

[26] Dopazo C, Martín J, Hierro J (2007). Local geometry of isoscalar surfaces. Phys. Rev. E.

[27] Trivedi S, Griffiths R, Kolla H, Chen J, Cant R (2019). Topology of pocket formation in turbulent premixed flames. Proc. Combust. Inst..

[28] Malkeson S, Chakraborty N (2010). statistical analysis of displacement speed in turbulent stratified flames: A direct numerical simulation study. Combust. Sci. Technol..

[29] Peters N (1999). The turbulent burning velocity for large-scale and small-scale turbulence. J. Fluid Mech..

[30] Cant, R. S. CUED/A-THERMO/TR67. Tech. Rep., Cambridge University Engineering Department (2012).

[31] Poinsot TJ, Lele SK (1992). Boundary conditions for direct simulations of compressible viscous flows. J. Comput. Phys..

[32] Rogallo, R. Numerical experiments in homogeneous turbulence. Tech. Rep. NASA-TM-81315 (1981).

[33] Batchelor G, Townsend A (1948). Decay of Turbulence in the Final Period. Proc. R. Soc. Lond. Ser. A.

[34] Eswaran V, Pope S (1988). Direct numerical simulations of the turbulent mixing of a passive scalar. Phys. Fluids.

[35] Doan N, Swaminathan N (2019). Analysis of markers for combustion mode and heat release in MILD combustion using DNS data. Combust. Sci. Technol..

